# A 21-Year-Old Pregnant Woman with Hypertension and Proteinuria

**DOI:** 10.1371/journal.pmed.1000037

**Published:** 2009-02-24

**Authors:** Andrea Luk, Ronald C. W Ma, Ching Wan Lam, Wing Hung Tam, Anthony W. I Lo, Enders K. W Ng, Alice P. S Kong, Wing Yee So, Chun Chung Chow

## Abstract

Ronald Ma and colleagues describe the differential diagnosis, investigation, and management of a 21-year-old pregnant woman presenting with hypertension and proteinuria at 20 weeks of gestation.

## DESCRIPTION of CASE

A 21-year-old pregnant woman, gravida 2 para 1, presented with hypertension and proteinuria at 20 weeks of gestation. She had a history of pre-eclampsia in her first pregnancy one year ago. During that pregnancy, at 39 weeks of gestation, she developed high blood pressure, proteinuria, and deranged liver function. She eventually delivered by emergency caesarean section following failed induction of labour. Blood pressure returned to normal post-partum and she received no further medical follow-up. Family history was remarkable for her mother's diagnosis of hypertension in her fourth decade. Her father and five siblings, including a twin sister, were healthy. She did not smoke nor drink any alcohol. She was not taking any regular medications, health products, or herbs.

At 20 weeks of gestation, blood pressure was found to be elevated at 145/100 mmHg during a routine antenatal clinic visit. Aside from a mild headache, she reported no other symptoms. On physical examination, she was tachycardic with heart rate 100 beats per minute. Body mass index was 16.9 kg/m^2^ and she had no cushingoid features. Heart sounds were normal, and there were no signs suggestive of congestive heart failure. Radial-femoral pulses were congruent, and there were no audible renal bruits.

Baseline laboratory investigations showed normal renal and liver function with normal serum urate concentration. Random glucose was 3.8 mmol/l. Complete blood count revealed microcytic anaemia with haemoglobin level 8.3 g/dl (normal range 11.5–14.3 g/dl) and a slightly raised platelet count of 446 × 10^9^/l (normal range 140–380 × 10^9^/l). Iron-deficient state was subsequently confirmed. Quantitation of urine protein indicated mild proteinuria with protein:creatinine ratio of 40.6 mg/mmol (normal range <30 mg/mmol in pregnancy).

### What Were Our Differential Diagnoses?

An important cause of hypertension that occurs during pregnancy is pre-eclampsia. It is a condition unique to the gravid state and is characterised by the onset of raised blood pressure and proteinuria in late pregnancy, at or after 20 weeks of gestation [1]. Pre-eclampsia may be associated with hyperuricaemia, deranged liver function, and signs of neurologic irritability such as headaches, hyper-reflexia, and seizures. In our patient, hypertension developed at a relatively early stage of pregnancy than is customarily observed in pre-eclampsia. Although she had proteinuria, it should be remembered that this could also reflect underlying renal damage due to chronic untreated hypertension. Additionally, her electrocardiogram showed left ventricular hypertrophy, which was another indicator of chronicity.

While pre-eclampsia might still be a potential cause of hypertension in our case, the possibility of pre-existing hypertension needed to be considered. Box 1 shows the differential diagnoses of chronic hypertension, including essential hypertension, primary hyperaldosteronism related to Conn's adenoma or bilateral adrenal hyperplasia, Cushing's syndrome, phaeochromocytoma, renal artery stenosis, glomerulopathy, and coarctation of the aorta.

**Box 1: Causes of Hypertension in Pregnancy**

- Pre-eclampsia
- Essential hypertension
- Renal artery stenosis
- Glomerulopathy
- Renal parenchyma disease
- Primary hyperaldosteronism (Conn's adenoma or bilateral adrenal hyperplasia)
- Cushing's syndrome
- Phaeochromocytoma
- Coarctation of aorta
- Obstructive sleep apnoea

Renal causes of hypertension were excluded based on normal serum creatinine and a bland urinalysis. Serology for anti-nuclear antibodies was negative. Doppler ultrasonography of renal arteries showed normal flow and no evidence of stenosis. Cushing's syndrome was unlikely as she had no clinical features indicative of hypercortisolism, such as moon face, buffalo hump, violaceous striae, thin skin, proximal muscle weakness, or hyperglycaemia. Plasma potassium concentration was normal, although normokalaemia does not rule out primary hyperaldosteronism. Progesterone has anti-mineralocorticoid effects, and increased placental production of progesterone may mask hypokalaemia. Besides, measurements of renin activity and aldosterone concentration are difficult to interpret as the renin-angiotensin-aldosterone axis is typically stimulated in pregnancy. Phaeochromocytoma is a rare cause of hypertension in pregnancy that, if unrecognised, is associated with significant maternal and foetal morbidity and mortality. The diagnosis can be established by measuring levels of catecholamines (noradrenaline and adrenaline) and/or their metabolites (normetanephrine and metanephrine) in plasma or urine.

### What Was the Diagnosis?

Catecholamine levels in 24-hour urine collections were found to be markedly raised. Urinary noradrenaline excretion was markedly elevated at 5,659 nmol, 8,225 nmol, and 9,601 nmol/day in repeated collections at 21 weeks of gestation (normal range 63–416 nmol/day). Urinary adrenaline excretion was normal. Pregnancy may induce mild elevation of catecholamine levels, but the marked elevation of urinary catecholamine observed was diagnostic of phaeochromocytoma. Conditions that are associated with false positive results, such as acute myocardial infarction, congestive heart failure, acute cerebrovascular event, withdrawal from alcohol, withdrawal from clonidine, and cocaine abuse, were not present in our patient.

The working diagnosis was therefore phaeochromocytoma complicating pregnancy. Magnetic resonance imaging (MRI) of neck to pelvis, without gadolinium enhancement, was performed at 24 weeks of gestation. It showed a 4.2 cm solid lesion in the mid-abdominal aorto-caval region, while both adrenals were unremarkable. There were no ectopic lesions seen in the rest of the examined areas. Based on existing investigation findings, it was concluded that she had extra-adrenal paraganglioma resulting in hypertension.

### What Was the Next Step in Management?

At 22 weeks of gestation, the patient was started on phenoxybenzamine titrated to a dose of 30 mg in the morning and 10 mg in the evening. Propranolol was added several days after the commencement of phenoxybenzamine. Apart from mild postural dizziness, the medical therapy was well tolerated during the remainder of the pregnancy. In the third trimester, systolic and diastolic blood pressures were maintained to below 90 mmHg and 60 mmHg, respectively. During this period, she developed mild elevation of alkaline phosphatase ranging from 91 to 188 IU/l (reference 35–85 IU/l). However, liver transaminases were normal and the patient had no seizures. Repeated urinalysis showed resolution of proteinuria. At 38 weeks of gestation, the patient proceeded to elective caesarean section because of previous caesarean section, and a live female baby weighing 3.14 kg was delivered. The delivery was uncomplicated and blood pressure remained stable.

### Progress

**Figure 1:**
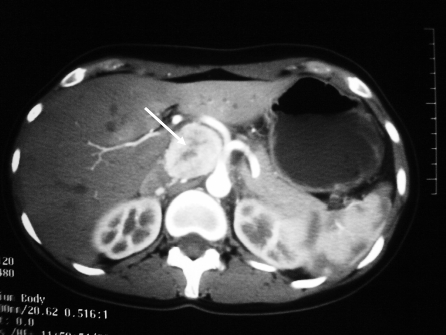
Computer Tomography Revealing an Extra-Adrenal Paraganglioma in the Celiac Region, Measuring 4.5 cm in Diameter

**Figure 2:**
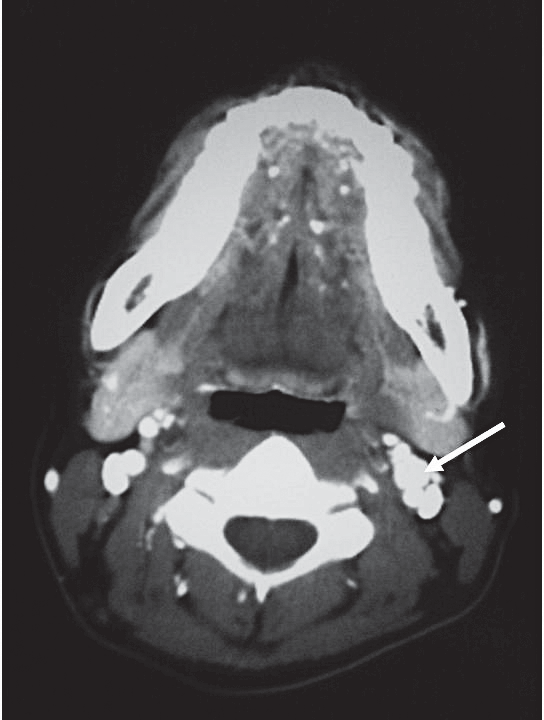
Computer Tomography Showing a 9 mm Well-Defined Hypervascular Nodule Present in Left Carotid Bifurcation, Suggestive of a Carotid Body Tumour

Following the delivery, computer tomography (CT) scan of neck, abdomen, and pelvis was performed as part of pre-operative planning to better delineate the relationship of the tumour to neighbouring structures. In addition to the previously identified extra-adrenal paraganglioma in the abdomen (Figure 1), the CT revealed a 9 mm hypervascular nodule at the left carotid bifurcation, suggestive of a carotid body tumour (Figure 2). The patient subsequently underwent an iodine (I)^131^ metaiodobenzylguanidine (MIBG) scan, which demonstrated marked MIBG-avidity of the paraganglioma in the mid-abdomen. The reported left carotid body tumour, however, did not demonstrate any significant uptake. This could indicate either that the MIBG scan had poor sensitivity in detecting a small tumour, or that the carotid body tumour was not functional.

**Figure 3:**
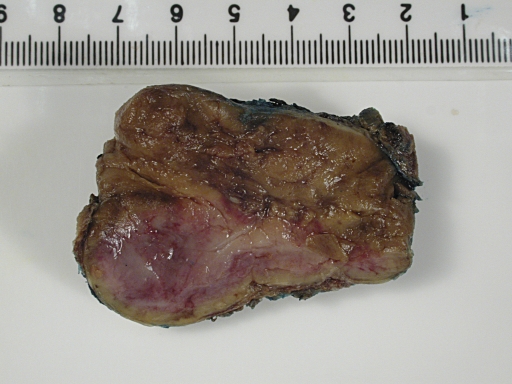
Macroscopic Appearance of the Resected Para-Aortic Mass The tumour is a well-circumscribed fleshy yellowish mass with maximal dimension of 5.5 cm.

**Figure 4:**
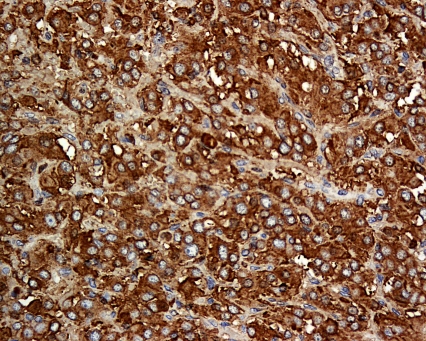
Histological Section of the Resected Tumour The tumour cells are polygonal with bland nuclei. The cells are arranged in nests and are immunoreactive to chromogranin (shown here) and synaptophysin.

In June 2008, four months after the delivery, the patient had a laparotomy with removal of the abdominal paraganglioma. The operation was uncomplicated. There was no wide fluctuation of blood pressures intra- and postoperatively. Phenoxybenzamine and propranolol were stopped after the operation. Histology of the excised tumour was consistent with paraganglioma with cells staining positive for chromogranin (Figures 3 and 4) and synaptophysin. Adrenal tissues were notably absent.

**Figure 5:**
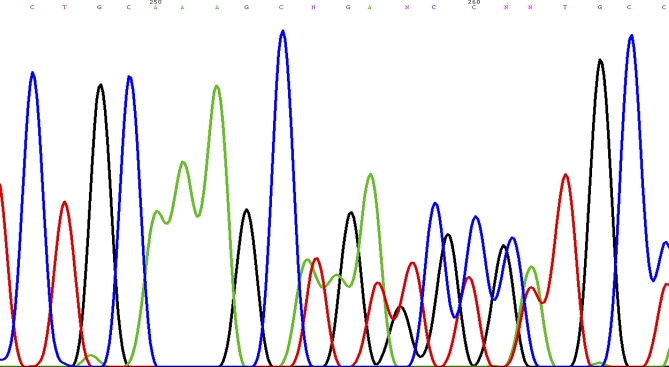
Sequence Chromatogram Showing the Novel Frameshift Mutation of the *SDHD* Gene: c.449_453dup (p.Val153LysfsX17)

The patient was counselled for genetic testing for hereditary phaeochromocytoma/paraganglioma. She was found to be heterozygous for c.449_453dup mutation of the *succinate dehydrogenase subunit D (SDHD)* gene (Figure 5). This mutation is a novel frameshift mutation, and leads to SDHD deficiency (GenBank accession number: 1162563). At the latest clinic visit in August 2008, she was asymptomatic and normotensive. Measurements of catecholamine in 24-hour urine collections had normalised. Resection of the left carotid body tumour was planned for a later date. She was to be followed up indefinitely to monitor for recurrences. She was also advised to contact family members for genetic testing. Our patient gave written consent for this case to be published.

## DISCUSSION

### Phaeochromocytoma in Pregnancy

Hypertension during pregnancy is a frequently encountered obstetric complication that occurs in 6%–8% of pregnancies [2]. Phaeochromocytoma presenting for the first time in pregnancy is rare, and only several hundred cases have been reported in the English literature. In a recent review of 41 cases that presented during 1988 to 1997, maternal mortality was 4% while the rate of foetal loss was 11% [3]. Antenatal diagnosis was associated with substantial reduction in maternal mortality but had little impact on foetal mortality. Further, chronic hypertension, regardless of aetiology, increases the risk of pre-eclampsia by 10-fold [1].

Classically, patients with phaeochromocytoma present with spells of palpitation, headaches, and diaphoresis [4]. Hypertension may be sustained or sporadic, and is associated with orthostatic blood pressure drop because of hypovolaemia and impaired vasoconstricting response to posture change. During pregnancy, catecholamine surge may be triggered by pressure from the enlarging uterus and foetal movements. In the majority of cases, catecholamine-secreting tumours develop in the adrenal medulla and are termed phaeochromocytoma. Ten percent of tumours arise from extra-adrenal chromaffin tissues located in the abdomen, pelvis, or thorax to form paraganglioma that may or may not be biochemically active. The malignant potential of phaeochromocytoma or paraganglioma cannot be determined from histology and is inferred by finding tumours in areas of the body not known to contain chromaffin tissues. The risk of malignancy is higher in extra-adrenal tumours and in tumours that secrete dopamine.

### Making the Correct Diagnosis

The diagnosis of phaeochromocytoma requires a combination of biochemical and anatomical confirmation. Catecholamines and their metabolites, metanephrines, can be easily measured in urine or plasma samples. Day collection of urinary fractionated metanephrine is considered the most sensitive in detecting phaeochromocytoma [5]. In contrast to sporadic release of catecholamine, secretion of metanephrine is continuous and is less subjective to momentary stress. Localisation of tumour can be accomplished by either CT or MRI of the abdomen [6]. Sensitivities are comparable, although MRI is preferable in pregnancy because of minimal radiation exposure. Once a tumour is identified, nuclear medicine imaging should be performed to determine its activity, as well as to search for extra-adrenal diseases. I^131^ or I^123^ MIBG scan is the imaging modality of choice. Metaiodobenzylguanidine structurally resembles noradrenaline and is concentrated in chromaffin cells of phaeochromocytoma or paraganglioma that express noradrenaline transporters. Radionucleotide imaging is contraindicated in pregnancy and should be deferred until after the delivery.

### Treatment Approach

Upon confirming the diagnosis, medical therapy should be initiated promptly to block the cardiovascular effects of catecholamine release. Phenoxybenzamine is a long-acting non-selective alpha-blocker commonly used in phaeochromocytoma to control blood pressure and prevent cardiovascular complications [7]. The main side-effects of phenoxybenzamine are postural hypotension and reflex tachycardia. The latter can be circumvented by the addition of a beta-blocker. It is important to note that beta-blockers should not be used in isolation, since blockade of ß2-adrenoceptors, which have a vasodilatory effect, can cause unopposed vasoconstriction by a1-adrenoceptor stimulation and precipitate severe hypertension. There is little data on the safety of use of phenoxybenzamine in pregnancy, although its use is deemed necessary and probably life-saving in this precarious situation.

The definitive treatment of phaeochromocytoma or paraganglioma is surgical excision. The timing of surgery is critical, and the decision must take into consideration risks to the foetus, technical difficulty regarding access to the tumour in the presence of a gravid uterus, and whether the patient's symptoms can be satisfactorily controlled with medical therapy [8,9]. It has been suggested that surgical resection is reasonable if the diagnosis is confirmed and the tumour identified before 24 weeks of gestation. Otherwise, it may be preferable to allow the pregnancy to progress under adequate alpha- and beta-blockade until foetal maturity is reached. Unprepared delivery is associated with a high risk of phaeochromocytoma crisis, characterised by labile blood pressure, tachycardia, fever, myocardial ischaemia, congestive heart failure, and intracerebral bleeding.

Patients with phaeochromocytoma or paraganglioma should be followed up for life. The rate of recurrence is estimated to be 2%–4% at five years [10]. Assessment for recurrent disease can be accomplished by periodic blood pressure monitoring and 24-hour urine catecholamine and/or metanephrine measurements.

### Genetics of Phaeochromocytoma

Approximately one quarter of patients presenting with phaeochromocytoma may carry germline mutations, even in the absence of apparent family history [11]. The common syndromes of hereditary phaeochromocytoma/paraganglioma are listed in Box 2. These include Von Hippel-Lindau syndrome, multiple endocrine neoplasia type 2, neurofibromatosis type 1, and *succinate dehydrogenase (SDH)* gene mutations. Our patient has a novel frameshift mutation in the *SDHD* gene located at Chromosome 11q. SDH is a mitochondrial enzyme that is involved in oxidative phosphorylation. Characteristically, *SDHD* mutation is associated with head or neck non-functional paraganglioma, and infrequently, sympathetic paraganglioma or phaeochromocytoma [12]. Tumours associated with *SDHD* mutation are rarely malignant, in contrast to those arisen from mutation of the *SDHB* gene. Like all other syndromes of hereditary phaeochromocytoma, *SDHD* mutation is transmitted in an autosomal dominant fashion. However, not all carriers of the *SDHD* mutation develop tumours, and inheritance is further complicated by maternal imprinting in gene expression. While it may not be practical to screen for genetic alterations in all cases of phaeochromocytoma, most authorities advocate genetic screening for patients with positive family history, young age of tumour onset, co-existence with other neoplasms, bilateral phaeochromocytoma, and extra-adrenal paraganglioma. The confirmation of genetic mutation should prompt evaluation of other family members.

**Box 2: Hereditary Phaeochromocytoma/Paraganglioma Syndromes**

- Von Hippel-Lindau syndrome
- Multiple endocrine neoplasia type 2A and type 2B
- Neurofibromatosis type 1
- Mutation of *SDHB*, *SDHC*, *SDHD*
- Ataxia-telangiectasia
- Tuberous sclerosis
- Sturge-Weber syndrome

**Key Learning Points**

- Hypertension complicating pregnancy is a commonly encountered medical condition.
- Pre-existing chronic hypertension must be considered in patients with hypertension presenting in pregnancy, particularly if elevation of blood pressure is detected early during pregnancy or if persists post-partum.
- Secondary causes of chronic hypertension include renal artery stenosis, renal parenchyma disease, primary hyperaldosteronism, phaeochromocytoma, Cushing's syndrome, coarctation of the aorta, and obstructive sleep apnoea.
- Phaeochromocytoma presenting during pregnancy is rare but carries high rates of maternal and foetal morbidity and mortality if unrecognised.
- Successful outcomes depend on early disease identification, prompt initiation of alpha- and beta-blockers, carefully planned delivery, and timely resection of the tumour.

### Conclusion

Phaeochromocytoma complicating pregnancy is uncommon. Nonetheless, in view of the potential for catastrophic consequences if unrecognised, a high index of suspicion and careful evaluation for secondary causes of hypertension is of utmost importance. Blood pressure should be monitored in the post-partum period and persistence of hypertension must be thoroughly investigated.

## Abbreviations

CT: computer tomography
I: iodine
MIBG: metaiodobenzylguanidine
MRI: magnetic resonance imaging
SDH: succinate dehydrogenase
SDHD: succinate dehydrogenase subunit D
